# Rabbit Haemorrhagic Disease Virus 2 (RHDV2; GI.2) in Ireland Focusing on Wild Irish Hares (*Lepus timidus hibernicus*): An Overview of the First Outbreaks and Contextual Review

**DOI:** 10.3390/pathogens11030288

**Published:** 2022-02-24

**Authors:** Andrew W. Byrne, Ferdia Marnell, Damien Barrett, Neil Reid, Robert E. B. Hanna, Máire C. McElroy, Mícheál Casey

**Affiliations:** 1 One-Health Scientific Support Unit, Department of Agriculture, Food and the Marine, Agriculture House, D02 WK12 Dublin, Ireland; damien.barrett@agriculture.gov.ie; 2 Department of Housing, Local Government and Heritage, National Parks and Wildlife Service (NPWS), D07 N7CV Dublin, Ireland; ferdia.marnell@housing.gov.ie; 3 Institute of Global Food Security (IGFS), School of Biological Sciences, Queen’s University Belfast, Belfast BT9 5DL, UK; neil.reid@qub.ac.uk; 4 Veterinary Science Division (VSD), Agri-Food and Biosciences Institute, Stormont, Belfast BT4 3SD, UK; bob.hanna@afbini.gov.uk; 5 Bacteriology and Parasitology Division, Department of Agriculture, Food and the Marine, Agriculture House, Backweston, W23 VW2C Dublin, Ireland; maire.mcelroy@agriculture.gov.ie; 6 Regional Veterinary Laboratories (RVL) Division, Department of Agriculture, Food and the Marine, Agriculture House, Backweston, W23 VW2C Dublin, Ireland; micheal.casey@agriculture.gov.ie

**Keywords:** rabbit haemorrhagic disease, *Lepus*, wildlife disease, wildlife infectious disease, lagovirus, wild–domestic interface, spillover infection

## Abstract

Rabbit haemorrhagic disease virus 2 (RHDV2; GI.2) is a pathogenic lagovirus that emerged in 2010, and which now has a global distribution. Outbreaks have been associated with local population declines in several lagomorph species, due to rabbit haemorrhagic disease (RHD)-associated mortality raising concerns for its potential negative impact on threatened or vulnerable wild populations. The Irish hare (*Lepus timidus hibernicus*) is endemic to Ireland, and is of conservation interest. The first cases of RHDV2 in Ireland were reported in domestic rabbits (*Oryctolagus cuniculus*) in 2016, soon followed by the first known case in a wild rabbit also in 2016, from a population reported to be experiencing high fatalities. During summer 2019, outbreaks in wild rabbits were confirmed in several locations throughout Ireland. Six cases of RHDV2 in wild hares were confirmed between July and November 2019, at four locations. Overall, 27 cases in wildlife were confirmed in 2019 on the island of Ireland, with a predominantly southern distribution. Passive surveillance suggests that the Irish hare is susceptible to lethal RHDV2 infection, and that spillover infection to hares is geographically widespread in eastern areas of Ireland at least, but there is a paucity of data on epidemiology and population impacts. A literature review on RHD impact in closely related *Lepus* species suggests that intraspecific transmission, spillover transmission, and variable mortality occur in hares, but there is variability in reported resistance to severe disease and mortality amongst species. Several key questions on the impact of the pathogen in Irish hares remain. Surveillance activities throughout the island of Ireland will be important in understanding the spread of infection in this novel host.

## 1. Introduction

Rabbit haemorrhagic disease virus 2 (RHDV2; GI.2) is a pathogen of concern for species in the order Lagomorpha, and has spread rapidly on a global scale, impacting local population dynamics and ecosystems via disease (RHD)-induced mortalities [1,2,3]. The emergence and spread of this virus are particularly concerning, given that a quarter of lagomorph species evaluated by the International Union for Conservation of Nature (IUCN) are considered to be threatened [4]. Some of the most vulnerable populations are isolated island endemics, which may be of particular concern. In this paper, we briefly review the pathogen and key aspects of its epidemiology, and present data on the initial known outbreaks within an endemic subspecies of mountain hare (*Lepus timidus*) that is unique to Ireland, the Irish hare (*L.t. hibernicus)*. The emergence of this infection in wild Irish hare populations is of concern from both conservation and wildlife management perspectives. This study was developed from research undertaken to inform evidence-based policy during the early period of the outbreak. 

The study illuminates the challenges of working on recently emerged infectious pathogens in a novel wildlife host population, where data are limited and the judicious extrapolation of evidence from other closely related species must be considered when informing policy. 

### 1.1. The Pathogen and Its Close Relatives

The aetiological agent that causes Rabbit haemorrhagic disease (RHD) is a non-enveloped single-stranded positive-sense RNA virus belonging to the genus *Lagovirus*, within the family of *Caliciviridae* [5]. Le Pendu et al. [6] have proposed, based on a phylogenetic analysis of the VP60 capsid gene only, that one species resides within the genus, *Lagovirus europaeus.* This species is split into two genogroups—GI, which includes the pathogens that cause RHD, and GII, which includes the pathogen that causes European brown hare syndrome (EBHSV; GII.1). The genogroup GI has four genotype members, of which GI.1 is the ‘classical’ strain (RHDV1) and GI.2 is the recent emergent strain (RHDV2; [6]) that cause RHD. 

The novel GI.2/RHDV2 was first recognised in Europe (France) in 2010 [1]. While GI.1/RHDV1 and GII.1/EBHSV are broadly animal genus-specific—GI.1/RHDV1 to rabbits, GII.1/EBHSV to hare species, and the eastern cottontail (*Sylvilagus floridanus* [7])-GI.2/RHDV2 has a broader host range [8]. GI.2/RHDV2 is known to infect both rabbits (*Oryctolagus cuniculus*) and hare (*Lepus*) species, including European (*L. europaeus*), mountain (*L. timidus*), Irish (*L.t. hibernicus*), Cape (*L. capensis subsp. mediterraneus*), Iberian (*L. granatensis*) and Italian (*L. corsicanus*) hares, black-tailed jackrabbit (*L. californicus*), and antelope jackrabbit (*L. alleni*) [9,10,11,12,13,14]. RHDV2 has been reported to infect several *Sylvilagus* (cottontail) species also, including eastern (*S. floridanus*)*,* desert (*S. audubonii*), and mountain (*S. nuttallii*) cottontails [13,15,16]. The GI.1/RHDV1 affects adult lagomorphs only, however, GI.2/RHDV2 viral infection has been shown to kill kits (as young as 11 days) as well as adults [1,3,10]. For simplicity in the rest of the text, we refer to RHDV1 or RHDV2, respectively.

The distribution of RHDV2 has expanded quickly on a global scale [3,16,17,18] since the pathogen was first discovered in France. In several populations, RHDV2 has taken over as the main RHD virus circulating in wild host populations [1,3,8,19].

### 1.2. Disease, Clinical Signs and Pathology

The *Lagoviruses* RHDV1 and RHDV2 are not believed to have any implications for humans (i.e., not zoonotic). They are highly pathogenic viruses of lagomorphs, with high RHD-associated mortality rates [20]. Historically, RHD was first discovered in China in 1984, in Angora rabbits imported from Germany infected with the classical RHDV1 [21]. The first European record of RHDV1 causing RHD was in Italy in 1986, and it quickly spread within Europe, and to the United States and Africa [22]. RHDV1 was trialled as a biocontrol agent of rabbits on an Australian island (Wardang) in 1991, but was accidentally introduced to the mainland, where spread was rapid, leading to declines in rabbit populations [23]. The pathogen remains a major part of the control strategy for invasive rabbits in Australia and New Zealand [24,25,26]. Two RHDV1 strains are approved for use there: (GI.1cP-GI.1c (v351 Czech) and GI.1aP-GI.1a (RHDVa-K5)), but not RHDV2, for deliberate release or for biocontrol purposes. In combination with the emergence of RHDV2 and its rapid spread [3], RHD now has a global distribution (with the exception of Antarctica) and is considered endemic in a number of countries, especially within wild European rabbit (*O. cuniculus*) populations. 

There are few detailed pathology studies specifically on RHDV2 [13,20,27,28,29], but in many aspects, disease progression resembles that induced by RHDV1 (in rabbits; [13]) and EBHSV (in hares; [8,11,27,30]. Infected animals develop fever (pyrexia), and antemortem clinical signs can also include anorexia, collapse, lethargy, seizures, icterus, bleeding from the mouth, dyspnoea, hypothermia, bradycardia, or poor blood clotting [28]. Seizures may be more frequent near death [20]. In the small number of cases where clinical signs have been described before death, these included epistaxis, apathy/depression, anorexia, and neurological signs such as ataxia and circling [12]. Pathologically, the infection is characterised by necrotising hepatitis, with gross morphological impacts on liver and spleen, haemorrhage in other tissues, and occasionally jaundice (icterus). The distinguishing diagnostic feature is massive hepatic necrosis [13], which has been consistently found in reports on the pathology of RHDV2 in hares [11,14,31]. Similarly, in domestic rabbits, RHDV2 infection was associated with hepatocellular necrosis on microscopic examination in 100% (n = 185) of cases [28]. In terms of the ranking frequency of the occurrence of pathological signs, Lankton et al. [13] report on RHDV2 for North American lagomorphs that massive hepatocellular dissociation and necrosis or apoptosis, and pulmonary congestion were found in 100% of cases examined; epistaxis, edema in the lung, and haemorrhage in the lung were found in 92% of cases examined; and acute renal tubular injury in 38% of cases. 

Mortality from RHDV1 in rabbits has been estimated at 90% or more in susceptible populations, and progression is rapid; mortality from RHDV2 appears more variable relative to RHDV1, and depends on the strain evolution of RHDV2 since emergence (see below). Animals typically die within 48–96 hours of being exposed to the pathogen. The viral load is high in infected animals that succumb, based on the low Ct value observed by real-time reverse transcription–polymerase chain reaction (RT-PCR) (e.g., [32]). Death without clinical signs can occur. In domestic rabbits, Harcourt-Brown et al. [28] reported no macroscopic abnormalities in 42% of 185 rabbit haemorrhagic disease cases infected with RHDV2.

Data on RHDV2 in hares specifically are limited at present, but it appears to be less virulent in hares than in rabbits, with lower and more variable mortality rates reported in experimental studies [1,11,33]. 

### 1.3. Epidemiology and Transmission

Some of the epidemiology of RHDV2 must be inferred from RHDV1 research, as we are currently limited with regard to population level and longitudinal epidemiological studies on the newly emerged virus. RHDV1 is a highly transmissible infectious agent, with both direct and indirect (via fomites, e.g., grass, bedding, shoes, clothing) transmission routes. The pathogen can be maintained for prolonged periods: in inorganic substrates for up to a month, and up to three months in organic substrates (e.g., infected carcasses; [34]). The RHDV1 pathogen has been reported to survive freeze–thaw action in tissue suspensions [35]. The maintenance of infection within populations is thought to be related to the presence of carcasses, which can maintain viable pathogens for several months. The primary transmission route is oral, but other transmission routes have been suggested (including nasal, subcutaneous, intravenous, and intramuscular). Vectors may spread infection [36], with insects (diptera), humans, and birds all potentially facilitating viral spread [23]. It is possible for non-lagomorph mammalian hosts to carry RHD viruses, therefore, the possibility of other wildlife hosts being involved in maintaining infection cannot be ruled out completely (e.g., RHDV2 has been reported in voles (*Microtus*), shrews (*Crocidura*), badgers (*Meles*), mice (*Apodemus, Mus*), fox (*Vulpes vulpes*), Tasmanian devil (*Sarcophilus harrisii*), and Alpine musk deer (*Moschus sifanicus*); [37,38,39,40,41,42]). However, it is uncertain whether these species are merely mechanical vectors, or represent truly permissive hosts.

There is some evidence to suggest that density-dependent mechanisms may be a feature of the epidemiology of RHD virus in both rabbit and hare populations [23]. Fa et al. [43], using an individual-based model, suggested that the spread and maintenance of RHD was affected by host density. Similarly, studies have suggested that EBHS maintenance in hare populations may be dependent on population density. For example, Chiari et al. [44] suggested that higher hare densities in a study area in Italy (threshold: > 15 hares/km^2^) were associated with 3.3 times the risk of EBHS positivity, in comparison with lower density populations. Furthermore, a mathematical model by Salvioli et al. [45], also using data from Italy, found that populations with densities < 7 hares/km^2^ tended not to sustain EBHS infection. The R_0_ (the number of secondary infections that an infectious individual can cause, values > 1 indicate maintenance, values < 1 indicate pathogen extinction) increased from 1.03 to 2.15 when density increased from 7 hares/km^2^ to 50 hares/km^2^.

There is some evidence to suggest that stress could be a contributing factor in increasing animal–animal transmission rates, at least in terms of rabbits and RHDV1 [46]. Schirrmeier et al. [47] also speculate that reproductive stress could impact host susceptibility, while Henzell et al. [48] suggested that co-infection (e.g., parasitic burden) and stress could interact to affect the recurrence of RHDV1 in rabbit populations in Australia. Furthermore, Cooke [46] suggested that the movement of carrier animals across populations may be required for the maintenance of RHDV1 infection. Recent studies have suggested that RHDV1 and RHDV2 spread may be facilitated by human activities [3,49,50]. Adán et al. [49] have suggested that there was no statistical difference in the mean spread rate reported for RHDV1 (330 km/year) relative to RHDV2 (479 km/year), reported in the scientific literature that they reviewed.

RHDV2 has recently been reported to be “responsible for a large proportion of lagovirus disease cases in hare populations in France” [8]. A similar pattern has been described in several other countries [19,51,52]. The reasons for this competitive advantage may be multifactorial, and include RHDV2 being capable of partially overcoming RHDV1 host immunity, where it exists [53], that RHDV2 has a wider host breadth [54], and the additional ability to cause disease in younger cohorts relative to RHDV1 [55,56]. Taggart et al. [56] identified the latter characteristic of RHDV2 as particularly important, as clinical infections of kittens can lead to high viral replication, shedding into the environment, and mortality. As RHDV1 is subclinical in young rabbits, the RHDV2-induced mortality of kittens lowers the susceptible adult population available for RHDV1 to infect [56].

Lagoviruses exhibit rapid evolution, typical of RNA viruses generally [57]. This may be for several hypothetical reasons, including an evolutionary arms race versus the host immune system, variation in host genetic susceptibility/infectivity/resistance, etc. [25]. This is some evidence from Sweden and Australia to suggest that, even over short periods of the epidemic detectable RHDV2 strain, variation has emerged with differing phenotypic characteristics [10,57]. In Sweden, it was reported that viral strains sampled early in the epidemic used in inoculations studies were less pathogenic than later strains [10]. It has been speculated that the selection pressure on the pathogen is favouring increased virulence, as evidenced by an experimental infection study of rabbits using a newer Australian strain of RHDV2, whereby death or severe disease requiring euthanasia occurred within 96 hours post-infection for all study animals [27], equivalent to classical RHDV1 pathogenicity. RHDV2 appears to have increased diversity relative to RHDV1, partly due to the recognised recombinant events with pathogenic and non-pathogenic viruses [57,58]. Capucci et al. [59] also suggested that more virulent strains of RHDV2 have emerged over time since emergence, based on the mortality in experimental conditions in rabbits inoculated with circulating strains from Italy in 2014 and 2015. They infer that the mortality rate of 80% was approximately four times higher than that found in the early RHDV2 studies undertaken prior to 2011. They conclude that “highly pathogenic RHDV2 strains have emerged during the virus’s evolution and have become prevalent in the field”. This speed of evolution and diversity complicates any assessment of the threat posed by these viruses—the threat is also diversifying and evolving. For example, Mahar et al. [57] reported at least six viable recombination events in a two-year period since the discovery of RHDV2 in mid-2015 in Australia [57,60]. However, recent experimental work from Australia suggests that in rabbits, the presence of maternal antibodies, from surviving exposed does to their kits, could provide protection from disease [29]. From the Australian perspective, this could impact RHDV2 as a biocontrol agent, but could have more beneficial interpretation elsewhere, where a reduction in disease-induced mortalities would be welcomed. However, the epidemiological situation is not simple, illustrated by the experimental work of Calvete et al. [61] in Spain. Spreading RHDV2 on baits during breeding periods was found to induce the infection of young rabbits and reduce mortality rates, presumably due to maternal antibody protection [61]. However, exposed young rabbits were also more susceptible to RHDV infection than control animals, leading to uncertainty as to how controlled exposure could be used to modulate the impact of RHD in wild populations, especially where several lagoviruses are circulating.

An additional issue is the lack of information with regard to the specific host–pathogen interaction, which is important regarding the Irish hare. Species-specific variation in the effects of RHD viruses in terms of host susceptibility has been reported [31,54]. Within species, genetic variation may also account for some of the variable reported resistance to infection across wild host populations [25,62].

### 1.4. Ecological Impact

Where rabbits are a keystone prey species, the incursion of RHD can have significant impacts on predator species [63]. In southern Spain, the emergence of RHD in rabbits caused by RHDV1 resulted in a 60–70% decline in population abundance in the 1980s–1990s [63]. This incursion of RHD in rabbits resulted in declines in Iberian lynx (*Lynx pardinus*) and the Imperial eagle (*Aquila heliaca*). It also caused a prey shift for other predators, for example the European badger (*Meles meles*), which had to rapidly adjust the composition of their diet in response to a 60–70% decline in rabbits in Doñana National Park [64]. By 2007, the highly virulent nature of the pathogen resulted in an estimated 250 million domestic and free-living rabbit deaths globally [65]. The arrival of RHDV2 has compounded reductions in rabbit densities in some monitored sites. RHDV2 has been associated with a declining population trajectory in several monitored populations of wild rabbits in Spain after its introduction [66]. In Doñana National Park, RHDV2 coincided with a precipitous decline in density (e.g., 3.24 rabbit/ha in 2009 to 0.16 rabbit/ha in 2013), despite restocking efforts [67]. Where rabbits and hares are invasive species, biological control via RHD can benefit local wildlife (e.g. [68,69]). 

In an Irish context, the rabbit is an important non-native but naturalised prey species (introduced during the 12th century) for several native and naturalised predators, e.g., the Irish stoat (*Mustela ermine hibernica*), fox (*Vulpes vulpes*), and buzzard (*Buteo buteo*; [70]), but the extent of food web impacts from rabbit declines due to RHD remain unknown. The potential ecological impacts resulting from disease in the Irish hare [12] remain unknown, but like the rabbit, it is also a key prey species for many predators, including reintroduced golden eagles (*Aquila chrysaetos)*.

### 1.5. Hosts in Ireland—Distribution, Abundance, and Autecology

The Irish hare is widespread throughout Ireland [70], with a low mean density of *ca*. 3 hares/km^2^, but substantial variation in the density of records reflecting spatial variation in density associated with habitat suitability [71]. The population exhibits significant interannual fluctuation [71], making population trends uncertain. The most recent estimate suggested a population of ca. 223,000 (111,000–449,000) individuals throughout the Republic of Ireland [71], with a further 41,000 (13,000–184,000) individuals throughout Northern Ireland [72]. There are two known populations of introduced, non-native, invasive European brown hares (*Lepus europaeus*) in Northern Ireland. 

Irish hares can breed throughout the year, with evidence that weather may impact species population dynamics [73]. Male hares become fertile during December, whilst females become fecund during January, initiating courtship and breeding [70]. Mountain hares have litters of 1–4 leverets, and females can produce 1–2 litters annually after their first year of life. Leveret mortality is high in the first year of life. The average reported longevity for mountain hares is 9 years, with only one small study reporting a minimum lifespan of 3–6.5 years for Irish hares.

Irish hares maintain home ranges that are larger in males (mean: 50ha; max: 70 ha), especially in winter, relative to females (mean: 21ha; max: 50ha; [72,74]). They can be vagile within these ranges, with daily movements exceeding 3km being reported [75]. Mountain hares tend not to disperse over great distances, and are highly philopatric to their natal area [74]. Data suggest that hares disperse over small scales of < 1km [74,76], but occasionally up to 12km [77], and genetic data from the Irish subspecies suggest that this may lead to population fragmentation [75]. Furthermore, there is some evidence to suggest sex-biased dispersal, with males exhibiting a greater likelihood to disperse from natal areas than females (i.e., females are more philopatric; [75]).

European rabbits are not native to Ireland, and were introduced in the 12th century [70]. The species is widespread and found in all counties, however, with some local absences notably in western regions where the predominant land cover is peat bog. The species appears to be more abundant in the south-east, however there is no national population estimate of rabbits in Ireland. The main breeding season is January to August, however, breeding can occur all year round. 

Rabbits and hares are sympatric across most of their ranges in Ireland [74]. They show very high levels of dietary overlap in grassland habitats, filling similar ecological niches, but probably with little direct competition [74]. The distribution and ecological niche overlap suggests that epidemics of shared infections could circulate between sympatric species, which has been reported elsewhere, and is a concern for spillover infection risk to Irish hares. Their widespread distribution, and lack of major barriers to dispersal, could facilitate the spread of infection across the island.

## 2. RHDV2 on the Island of Ireland (2016–2019)

The wild rabbit and hare carcasses examined and reported in this study were derived from reports of dead wild rabbits or hares notified by the public to Wildlife Conservation Rangers employed by the Irish National Parks and Wildlife Service (NPWS), the statutory body charged with the conservation of wildlife in Ireland. They were submitted to one of six Regional Veterinary Laboratories (RVLs), which are small multidisciplinary veterinary diagnostic and disease surveillance laboratories operated by the Irish Department of Agriculture, Food and the Marine, and are located in Athlone, Backweston (Dublin), Cork, Kilkenny and Sligo. Animal histories were taken from the submitting NPWS officer by the RVL and this information was entered, along with all other subsequent data, findings, and test results on the Department’s Laboratory Information Management System (LIMS). Each animal was subjected to a standard necropsy, which consisted of an external examination (noting any visible lesions or evidence of trauma), and the carcass was then opened and examined internally. Any gross lesions were recorded. A sample of liver was collected for RHDV1 and RHDV2 screening, and further samples were taken, and tests were performed based on the professional judgement and discretion of the veterinarian carrying out the necropsy. These additional examinations included microbiology, parasitology, and histopathology, and were conducted at the local site, or at the laboratory service headquarters in Backweston. For example, parasitological examination was not routinely done, but was requested if diarrhoea, gastroenteritis, or poor body condition was observed. Similar approaches were undertaken in laboratories in the Agri-food and Biosciences Institute (AFBI) for samples originating in Northern Ireland. The cases are presented in Table 1 and additional information on reported history, gross findings, histopathology and other conditions, where available, is presented in Supplementary Table S1. All cases were diagnosed using reverse transcription real-time–polymerase chain reaction (RT-qPCR); no sequencing was undertaken. Additional details of the laboratory procedures can be found in Kennedy et al. [12].

### 2.1. Domestic/Pet Animals

The first report of rabbits with RHDV2 in Ireland was in September 2016 [78]. In a report of the World Organisation for Animal Health (OIE) (2016), the first case with an RT-PCR positive result (Moredun Research Institute, UK) was a pet rabbit which had been vaccinated with a myxomatosis-RHD vaccine from an urban area in Co. Wicklow [79]. The rabbit had access to a garden, but otherwise had no known link to wild rabbits. The second case (Co. Clare) was an outbreak in six rabbits in a household, where four became sick and died. Infection was confirmed by RT-PCR. The surviving animals showed no clinical signs of infection. All animals in the second case had myxomatosis-RHD vaccination. There was no known epidemiological link between these initial cases.

**Figure 1:**
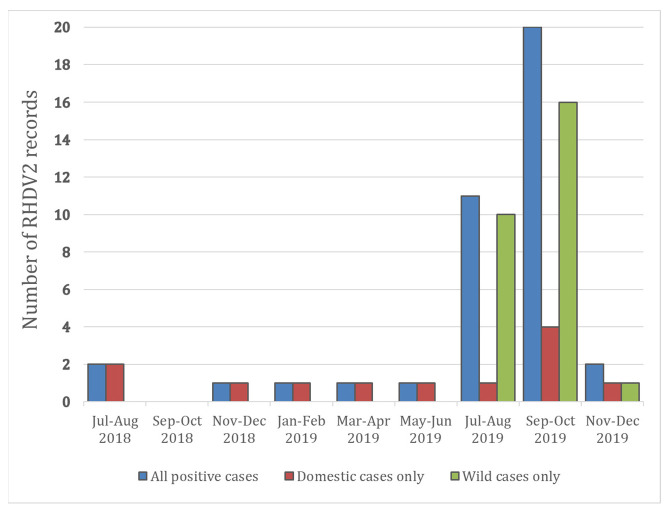
Frequency of RHDV2 cases reported from the island of Ireland during 2018–2019 for all (blue), domestic (red), and wild (green).

Further confirmed reports were catalogued within the Department of Agriculture, Food and the Marine (DAFM) Laboratory Information Management System (LIMS) database 2018–2019 (Figure 1; Table 1). Included were young kits: three 2.5-week-old kits, and one 9-week-old. 

**Figure 2:**
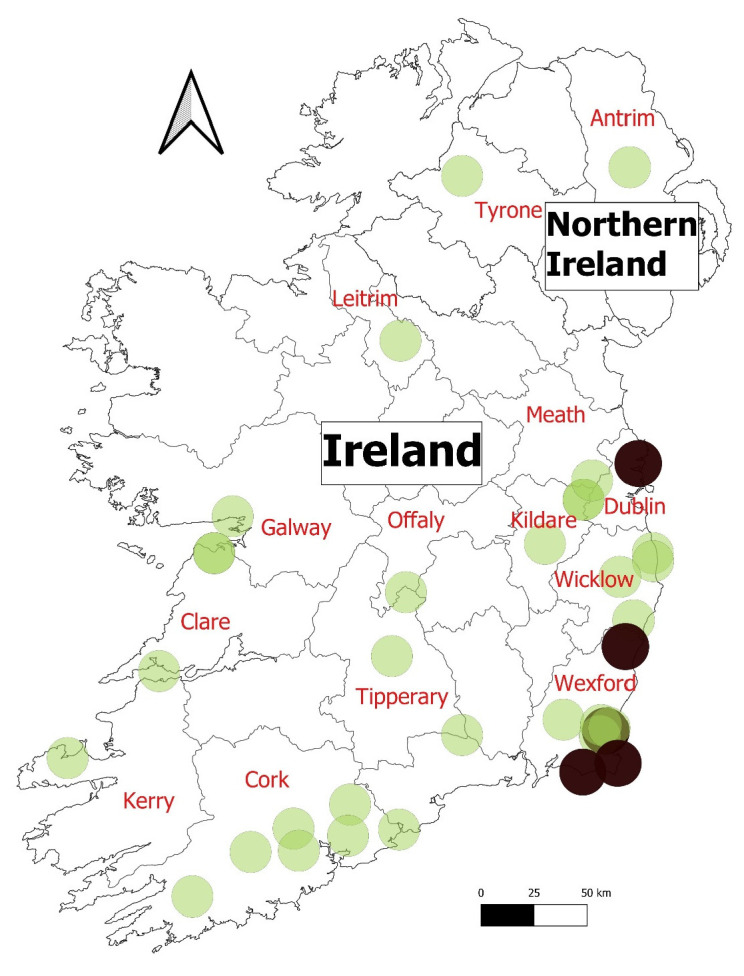
Distribution of RHDV2 cases during the study period on the island of Ireland. Cases in rabbits are green dots and Irish hare black.

There was a wide geographic distribution of these cases, across several different counties (Figure 2). No known epidemiological links were established between these cases. 

In Northern Ireland (UK), the first confirmed cases of RHDV2 were made in 2019 (R. Hanna, AFBI-NI, pers. com.) in domestic rabbits; there were two cases of RHDV2 confirmed by RT-PCR; one in Co. Tyrone (January), and a second in Co. Antrim (August). It should be noted that these two RHD cases were the only ones sent for confirmation (to Moredun Institute, UK) that year. Therefore, it is not possible to ascertain whether the pathogen was circulating in Northern Ireland before 2019 and was being diagnosed as RHDV1. 

### 2.2. Wild Animals

In 2016, a two-year-old wild rabbit was submitted to the Cork Regional Veterinary Laboratory. The carcass submitter described it as being one of a “high number of casualties in wild rabbits in Cork” [80]. This animal had “died suddenly” and exhibited non-specific pulmonary congestion, but no visible lesions in other organs were reported. RHDV2 was confirmed via RT-PCR at an RNA load considered to be evidence of causation. 

No further reports in wild rabbits were recorded until July 2019 (Figure 1), when a wild rabbit from Avoca in Co. Wicklow and another from Scattery Island in the Shannon Estuary (Co. Clare) were confirmed with RHDV2 infection (Figure 2). The animal from Scattery Island was one of several visibly sick and dying rabbits seen by the National Parks and Wildlife Service (NPWS) Conservation Ranger on the island at that time (NPWS pers. obs.). In July–August 2019, two Irish hares from Co. Wexford were submitted to Kilkenny Regional Veterinary Laboratory. They were diagnosed with viral haemorrhagic disease, and the causative agent was confirmed as RHDV2—these were the first cases of this virus in this subspecies [12]. One of these animals was from Wexford Wildfowl Reserve, an area known to have a high density of hares. 

These initial cases were followed by confirmed cases in wild rabbits in 10 counties (Clare, Leitrim, Cork, Kildare, Offaly, Wexford, Wicklow, Meath, Tipperary, Kerry; Figure 2) from August to November 2019. After the initial cases in wild hares, additional cases were confirmed in two counties, Wexford and Dublin. In Co. Wexford, infections were from multiple sites (minimum 20km between Gorey, Wexford Wildfowl Reserve, and Ballytrent). The clustering of records in the south-east of the island could suggest a hotspot of infection, however, it is uncertain as to whether this is a true epidemiological hotspot, or a sampling artefact.

No records of RHDV2 in wild animals were reported during the study period in Northern Ireland, with all RHD-diagnosed cases (by histopathology) being in domestic rabbits (n = 9 cases; 2016–2019).

Over 80% of all samples were taken at post-mortem from diseased animals, however, prevalence or mortality rates cannot be inferred from this study.

### 2.3. Summary of Laboratory Findings

Histories recorded about the individual (rabbit and Irish hare) cases reported several instances of high group (mean mortality: 86.5% (45/52 domestic rabbits); group range: 50–100%) or local mortality events (multiple fatalities referred to in n = 11 reports; see Supplementary Material Table S1). Gross pathological findings in lungs (n = 13), liver (n = 3), and kidneys (n = 3) were reported; primarily presenting with hyperaemia (n = 8), oedema (n = 3), and haemorrhages (n = 8). Hepatic necrosis was commonly reported (n = 22). Interstitial pneumonia was reported in five cases. *Staphylococcus* was isolated from a liver abscess in one case, and parasitological findings were reported in six cases (coccidia, n = 4), but only one case was described as “high parasite burden”. The primary pathological feature of RHDV2 infection in Irish hares specifically is the presence of massive acute or peracute hepatic necrosis. One Irish hare was found to have sinusoidal leukocytosis in the liver.

## 3. Ecological Risk to Irish Hares of the Emergence of RHDV2

The discovery of RHDV2 in Irish hares in 2019 was a significant cause for concern for the subspecies, which is of conservation and cultural importance. At the outset of these outbreaks, several key questions were required to be understood to evaluate the ecological risk to Irish hares of this emergent infection. Available evidence, which was used to inform these questions as much as possible, is presented below.

### 3.1. How Susceptible Are Hares to RHDV2?

The difference between RHDV2 and previously described lagoviruses (RHDV1, and EBHSV) is that it has increased age susceptibility (can kill kits/leverets, whereas RHDV1 and EBHSV cause fatalities in adult age classes only for rabbits and hares, respectively) and species range (i.e., extends across rabbits, several hare species/subspecies, and several *Sylvilagus* species). “In rabbits younger than 4–6 weeks, the RHDV…infection course is subclinical, but when the causative agent is RHDV2, clinical signs and mortality are observed even in young animals from 15 to 20 days of age onwards” [33].

Several species of hare have been found to be susceptible to RHDV2, with differing vulnerabilities, as discussed above. Camarda et al. [54] reported the transmission of RHDV2 to Italian hares (*L. corsicanus*) penned near to a rabbit enclosure (30-metre distance) which experienced a high mortality outbreak. On the basis of a low mortality rate (1 in 30 death rate), the authors speculated that the “virulence of RHDV2 is reduced in *L. corsicanus* when compared with the far higher mortality observed in *O. cuniculus*”. However, it is possible (though potentially less probable), to interpret their findings to suggest that the exposure of RHDV2 infectious dose (force of infection) to the hares was lower than the rabbit enclosure, due to the 30m separation between pens. Puggioni et al. [31] were the first to report outbreaks of RHDV2 in Sardinian Cape hare (*L. capensis mediterraneus*), and suggested that the pathogen “affected [both] rabbits and Cape hares, causing in both species a similar necrotising hepatitis with a comparable degree of mortality”. Importantly, given the data capture methodology, actual mortality rates could not be estimated during the study (i.e., there were neither detailed numerator nor denominator data). Puggioni et al. [31] also discuss early data (2010–2011) from France and Italy, where there were outbreaks of RHDV2, yet no European brown hare cases were reported. The authors speculate that this demonstrates that the European brown hare is less susceptible to RHDV2 than sympatric rabbits. However, there is evidence to suggest that RHDV2 strains became more pathogenic over time [10,57].

The number of cases of RHDV2 in hares in Ireland described during the study period was low, however, from an epidemiological perspective, this could not be a basis for inferring low susceptibility as: 1. There was no active monitoring scheme in endemic regions (only passive case detection); 2. Cases of mortality in hares have only been reported through passive surveillance. Only one peer reviewed paper reported the “ability of Mountain hares to serve as competent maintenance hosts for GI.2 in the absence of other leporids”, and suggested that more research is required [2].

### 3.2. Is Hare-to-Hare Transmission Possible?

Hare-to-hare transmission of RHDV2 appears to be possible [8], with the maintenance of infection in an island population of mountain hares (isolated from rabbits) in Sweden for a number of months being reported [2]. However, this assertion needs to be supported by experimental studies, as infection may have been maintained within the Swedish hare population by environmental contamination (indirect transmission) or by multiple incursions of infection into the population from the mainland (where there was a rabbit RHDV2 outbreak). Le Gall-Reculé et al. [8] speculate that the intra-specific and inter-specific transmission of infection were involved in the common (40% of cases in 2015 were RHDV2) widespread distribution cases reported in France.

There is limited evidence to suggest that hares have increased resistance (or susceptibility) to RHDV2, despite hare species being naturally resistant to the classical RHDV1 genotype [79]. There may be variation across hare species within Europe in terms of susceptibility, with the limited information available tentatively suggesting that European brown hares are most resistant, followed by the Italian hare, with the Sardinian cape hare being least resistant [54]. The outbreaks reported here in Irish hares are sporadic, though there appears to be some clustering of outbreaks in one county (Wexford). This could indicate some circulation of RHDV2 within local hare populations, but it is possible that these represent multiple spillover events from sympatric rabbit species that are also locally abundant.

### 3.3. Does RHDV2 Exhibit Lowered Virulence in Hares, Relative to Other RHD Strains and Hosts?

Hall et al. [81], in their initial report of RHDV2 in European brown hares in Australia, affirm that “the virulence and associated case fatality rate of RHDV2 in *L. europaeus* compared with *O. cuniculus*, and their susceptibility to infection, has not yet been determined”. While there may be some evidence to suggest the differential virulence of other related lagovirus pathogens between hosts, for example the species-specific mortality effects of RHDV1 on rabbits and the European brown hare syndrome virus (EBHSV; RHDV1 GII1; Lavazza et al. 1996), the RHDV2 strain has been shown to have fatal effects for both rabbits and hares [1,10,81]. 

In rabbits, experimental infection studies have demonstrated differences in virulence between classical RHDV and RHDV2, for example, Le Gall-Reculé et al. [1] reported later and more prolonged mortality events with RHDV2; more frequent subacute/chronic courses of infection with RHDV2; the frequent occurrence of severe liver degeneration and discoloration, splenomegaly and jaundice that characterise the subacute/chronic form of RHD; importantly, mortality rates were lower but more variable for RHDV2 relative to classical RHDV [1]. Mean mortality rates of 70–90% for RHDV1 and 5–70% for RHDV2 have been reported [33]. At least some of the variation in the reported mortality rates for RHDV2 may be related to host age and infectious dose [29]. However, recent experimental work suggests that the case fatality rate can reach 100% in rabbits [20,32]. Mohamed et al. [32] undertook a comparative experimental study on the effects of RHDV2 on wild caught eastern cottontail (*Sylvilagus floridanus*) relative to New Zealand white rabbits (NZWR), and demonstrated that cottontails had lower disease-induced mortality in comparison with NZWR.

The variation in mortality has been speculated to be modulated by “innate functionality of the immune response and/or its prompt activation by other pathogens” [82]. Capucci et al. [59] also speculate on the role that enteric bacterial coinfection could have on the course of RHDV2 infection, based on an apparent strong inhibition of viral replication at least 96 hrs post-inoculation during an experimental infection study. 

The pathological findings (see Supplementary Material) resulting from RHDV2 infection in Irish hares appears similar to that reported in other lagomorph species [13; 28; present study], with gross pathology reported in lung, liver and kidney tissue, and histopathology frequently revealing massive necrotising hepatitis. 

### 3.4. Are Hares a Reservoir of Infection?

In the Irish context, this is currently unknown. However, experiences in France, Italy, Sweden, Spain and elsewhere suggest that cases of RHDV2 in hare species correlate with known outbreaks in local rabbit populations (e.g., [2,8,11,83]). Molecular genotyping data have suggested an epidemiological association between a local rabbit outbreak in mainland Sweden and an isolated hare island population, though the mechanism of transmission to the island was undetermined (speculated to relate to human, insect, or bird movement [2]). Neimanis et al. [8] describe that “Once the virus arrived on the island, GI.2 circulated for at least 4.5 months based on necropsy findings, and six months based on observations of dead hares in the field, all in the absence of rabbits”. This suggests that hares may be able to maintain infection for periods of weeks to months in the absence of sympatric rabbit populations. Neimanis [10] debates whether RHDV2 was either introduced once to the island and then persisted for the duration of the outbreak, or it was repeatedly introduced. Velarde et al. [11] describe sporadic outbreaks of infection of RHDV2 in European brown hares in Italy and Spain, and conclude that hares in these populations are spillover hosts in situations where infection pressure from local infected rabbit populations is high. Hall et al. [81] also present data of the first cases in Australia of RHDV2 in hares, all of which were in close proximity to outbreaks in local rabbit populations.

Reports from Great Britain of hares infected with RHDV2 suggest a small number of hares succumbing to infection [84], which has been interpreted as “possibly representing a spill-over event from rabbits” [9]. However, the same authors suggest that the “impact of this infection on the Scottish brown hare population, which is already under pressure from changes in agricultural and wildlife management practices, can only be detrimental [to their conservation]”. Indeed, finding RHDV2 in Irish hares causing mortalities in at least three geographically separated populations is also a cause for concern for the Irish hare, especially given that the species is managed in many areas for legal hare coursing by local clubs [12]. Coursing involves the pursuit of an Irish hare by two competing muzzled greyhound dogs. Irish hares are gathered from local populations, held in ‘parks’ prior to coursing events, and thus increase the probability of direct or indirect interactions between conspecifics. Surviving hares are returned to the wild after the event. The outbreaks have led to mitigation measures and restrictions on licences for these activities [12].

### 3.5. Can Hares Transmit Infection into Other Host Populations (for Example, Rabbits)?

There currently appears to be little evidence to support or refute the hypothesis that hares can seed infection into other hosts. As the pathogen can infect other lagomorphs, in Ireland this could be rabbits or the small population of European hares, and transmission can occur via direct and indirect mechanisms; it is possible for ‘spillback infection’ (*sensu* [85]) to occur. Rabbit–hare infection has been inferred in a number of countries (e.g., Spain, Sweden, Australia, and Italy) using serological surveys and/or molecular epidemiological approaches sampling sympatric populations [8,10,31,54,81].

Given mean densities of Irish hares at ca. 3 hares/km^2^, and based on previous modelling work suggesting density dependence, the R_0_ of the disease may be < 1, and thus have a limiting spread. However, some populations can reach higher densities, e.g., 30 hares/km^2^ in Belfast International Airport. Many areas in the wider countryside are likely to have densities ca. 15 per km^2^ (N. Reid, pers com.). We may expect highly variable epidemic cycles depending on local ecological conditions. 

## 4. Conclusions

We have presented information on the RHDV2 situation in lagomorphs in Ireland from competent authority laboratories during early 2018–2019 outbreaks, with particular focus on presumed spillover to Irish hares, and in the context of international literature. Given our current understanding and available evidence, several assertions have been made regarding RHDV2 in Irish hare populations, but several significant evidence gaps have also been identified.

Hares, including Irish hares (*L. timidus hibernicus*), are susceptible to RHDV2 infection, and this has resulted in several mortalities primarily recorded in the southeast of Ireland. However, more data are required from populations exposed to the pathogen to assert whether the species exhibits low susceptibility. It is not possible to assert that hares are resistant *per se* to RHDV2, though there is evidence that relative resistance to succumbing to infection varies between different hare species (and potentially across populations within species). There are no data to infer where the Irish hare may be on this spectrum. Data from an island population of mountain hares in Sweden suggest that hare-to-hare transmission is possible, but maintenance in the absence of alternative hosts (i.e., rabbits) is currently uncertain, and may depend on ecological characteristics, including local densities and population sizes. It appears that RHDV2 may have reduced mortality impacts relative to RHDV1, though there is evidence to suggest that RHDV2 may have become more pathogenic since its emergence in 2010. Some authors have speculated that rabbits might be more sensitive to RHDV2 relative to hares, and that hares are not the primary host. Experimentally infected hares can succumb to disease-induced pathologies. Replication and transmission have occurred across the pathogen’s range primarily in rabbits, leading to the widespread replacement of RHDV1 with RHDV2 strains in wild populations, probably due to RHDV2 evading any host resistance built-up from exposure to RHDV1, being able to cause disease in younger cohorts than RHDV1, and because of its greater host range. It is currently unknown whether Irish hares represent a reservoir of infection or a dead-end spillover host in Ireland. Across the pathogen’s range, molecular epidemiology supports the contention that spillover from rabbit-to-hares occurs, however, whether spill-back occurs is uncertain. Future studies from Ireland could include investigating the molecular epidemiology of RHDV2 outbreaks, which may provide significant insights into shared strains in space, time and across host species. The considerable uncertainties identified during this study regarding RHDV2 in Irish hares suggest that further careful studies, including the population and disease surveillance activities of wild and managed (e.g., for coursing) populations, are warranted.

## Figures and Tables

**Table 1:** Confirmed cases of RHDV2 recorded in the Department of Agriculture, Food and Marine Laboratory Information System (DAFM-LIMS) database, and from the Agri-food and Biosciences Institute, Northern Ireland (AFBI-NI), for 2018–2019.

Case	Date	Laboratory	Pet/Wild	County	Animal Type	PM Known
1	20 August 2018	Limerick	Pet	Tipperary	Rabbit	Carcass
2	21 August 2018	Cork	Pet	Cork	Rabbit	Carcass
3	4 December 2018	GV	Pet	Wicklow	Rabbit	Diagnostic
4	25 January 2019	AFBI-NI	Pet	Tyrone	Rabbit	Carcass
5	15 April 2019	Cork	Pet	Cork	Rabbit	Carcass
6	20 June 2019	Cork	Pet	Cork	Rabbit	Carcass
7	10 July 2019	Limerick	Wild	Clare	Rabbit	Carcass
8	11 July 2019	GV	Wild	Wicklow	Rabbit	Diagnostic
9	25 July 2019	Kilkenny	Wild	Wexford	Hare	Carcass
10	12 August 2019	Limerick	Wild	Clare	Rabbit	Carcass
11	14 August 2019	Kilkenny	Wild	Wexford	Hare	Carcass
12	15 August 2019	Sligo	Wild	Leitrim	Rabbit	Carcass
13	26 August 2019	Limerick	Wild	Clare	Rabbit	Carcass
14	27 August 2019	Cork	Wild	Cork	Rabbit	Carcass
15	30 August 2019	Athlone	Wild	Kildare	Rabbit	Carcass
16	30 August 2019	Athlone	Wild	Offaly	Rabbit	Carcass
17	4 September 2019	Kilkenny	Wild	Wexford	Rabbit	Carcass
18	10 September 2019	GV	Pet	Kildare	Rabbit	Diagnostic
19	19 September 2019	Kilkenny	Pet	Tipperary	Rabbit	Carcass
20	26 September 2019	Dublin	Wild	Wicklow	Rabbit	Carcass
21	27 September 2019	Kilkenny	Wild	Wexford	Rabbit	Carcass
22	27 September 2019	Kilkenny	Wild	Wexford	Rabbit	Carcass
23	30 September 2019	Dublin	Wild	Kildare	Rabbit	Carcass
24	1 October 2019	Dublin	Wild	Dublin	Hare	Carcass
25	1 October 2019	Kilkenny	Wild	Wexford	Hare	Carcass
26	7 October 2019	Kilkenny	Wild	Wicklow	Rabbit	Carcass
27	8 October 2019	Dublin	Wild	Meath	Rabbit	Carcass
28	9 October 2019	Limerick	Wild	Tipperary	Rabbit	Carcass
29	16 October 2019	Cork	Wild	Cork	Rabbit	Carcass
30	17 October 2019	Kilkenny	Wild	Wexford	Rabbit	Carcass
31	17 October 2019	Athlone	Pet	Galway	Rabbit	Carcass
32	18 October 2019	Dublin	Wild	Wexford	Hare	Carcass
33	21 October 2019	GV	Pet	Unknown	Rabbit	Diagnostic
34	22 October 2019	Cork	Wild	Kerry	Rabbit	Carcass
35	23 October 2019	Kilkenny	Wild	Wexford	Rabbit	Carcass
36	29 October 2019	Kilkenny	Wild	Wexford	Hare	Carcass
37	1 November 2019	Cork	Wild	Cork	Rabbit	Carcass
38	29 November 2019	AFBI-NI	Pet	Antrim	Rabbit	Carcass
39	16 December 2019	Cork	Pet	Cork	Rabbit	Carcass

## Data Availability

All data are presented within the paper.

## Supplementary Materials

The following supporting information can be downloaded at: https://www.mdpi.com/article/10.3390/pathogens11030288/s1, Table S1: Details of recorded from a laboratory information management system (LIMS) of cases of RHDV2 in Ireland during outbreaks in 2018–2019.
