# Supplementary material for: Rabbit Haemorrhagic Disease Virus 2 (RHDV2; GI.2) in Ireland Focusing on Wild Irish Hares (Lepus timidus hibernicus): An Overview of the First Outbreaks and Contextual Review
Source: Pathogens. 2022 Feb 24;11(3):288. doi: 10.3390/pathogens11030288 (PMC8953227; doi:10.3390/pathogens11030288)
Supplement: Supplementary file 1 [file pathogens-11-00288-s001.zip › pathogens-1524979-supplementary.pdf]

**Supplementary Table S1:** Details of recorded from laboratory information management systems (LIMS) of cases of RHDV2 in Ireland and Northern Ireland during outbreaks in 2018–2019.

| I/D | Date of Submission | Species | Domestic/Wild | History | Gross PM findings | Histopathology | Other conditions (parasites?) |
|---|---|---|---|---|---|---|---|
| **Lime/18/02031** | 20/08/2018 | Rabbit | Domestic x 4 | 100% mortality in group of 23 rabbits; Short duration of illness (<1 day); Some nervous signs | Focal liver abscess in one rabbit | Liver: acute necrotising hepatitis | *Staphylococcus* spp. Isolated from liver abscess |
| **Cork/18/02665** | 21/08/2018 | Rabbit | Domestic | 9-week-old rabbit. Six of eight rabbits in litter have died. | Body condition: poor; Lungs: hyperaemia and oedema; | Liver: peracute to acute massive hepatic necrosis | Parasitology: coccidial oocysts detected in intestinal contents (significance undetermined) |
| **GVir/18/01633** | 04/12/2018 | Rabbit | Domestic | Diagnostic sample | Not applicable | Not applicable | |
| **2019–20022 (A)** | 25/01/2019 | Rabbit | Domestic | Sudden death. No signs noted | Pulmonary hyperaemia; | Liver: massive hepatocellular necrosis; periportal lymphocytic infiltration; Lung: necrosis of the vascular endothelium with lymphocytic infiltration of the perivascular spaces. | |
| **2019–20022 (B)** | 25/01/2019 | Rabbit | Domestic | Sudden death. No signs noted | Pulmonary hyperaemia; Kidneys: subcapsular petechial haemorrhages | Liver: periportal, necrotising hepatitis. Lung: necrosis of the vascular endothelium with lymphocytic infiltration of the perivascular spaces. | |

| | | | | | | | |
|---|---|---|---|---|---|---|---|
| **Cork/19/01277** | 15/04/2019 | Rabbit | Domestic | 14-week old rabbit displaying lethargy and progressing quickly to death. 13 animals have died in a group of 16. | Unremarkable | Liver: necrotising hepatitis | Parasitology: light coccidial burden in intestinal contents |
| **Cork/19/01892** | 20/06/2019 | Rabbit | Domestic | Two 4-5-month old rabbits. Sudden death. | Not reported | Not reported | |
| **Lime/19/01549** | 10/07/2019 | Rabbit | Wild | Found dead | Autolysed | Not applicable | |
| **GVir/19/00931** | 25/07/2019 | Rabbit | Wild | Diagnostic sample | Not applicable | Not applicable | |
| **Kilk/19/02024** | 25/07/2019 | Hare | Wild | Juvenile hare noticed running in circles before dropping dead | Lung: mild multifocal hyperaemia; large volume soft green large intestinal contents | Liver: Multifocal, random,necrotising hepatitis - acute. Spleen: depletion of red pulp. | |
| **Lime/19/01737** | 12/08/2019 | Rabbit | Wild | One of several visibly sick and dying rabbits seen by the National Parks and Wildlife Service (NPWS) Conservation Ranger on an island | Unknown | Unknown | |
| **Kilk/19/02157** | 14/08/2019 | Hare | Wild | Not provided | Lungs: mild hyperaemia; Kidneys: hyperaemia | Liver: peracute to acute, multifocal necrotising hepatitis; | |
| **Slig/19/03326** | 15/08/2019 | Rabbit | Wild | Found dead. Recent disappearance of rabbits in the locality - some noticed to be lethargic and not eating. | Autolysed. | Not applicable | |

| | | | | | | |
|---|---|---|---|---|---|---|
| **Lime/19/01831** | 26/08/2019 | Rabbit | Wild | Two rabbits found dead in close proximity. One scavenged and unsuitable for necropsy | Unremarkable. | Not applicable |
| **Cork/19/02403** | 27/08/2019 | Rabbit | Wild | Convulsing | Intestine: multifocal, serosal petechial haemorrhages | Liver: acute, severe, periportal, necrotising hepatitis; Kidney: multifocal haemorrhages; Heart: multifocal myocardial haemorrhages; Unremarkable |
| **Athl/19/01799 (1)** | 30/08/2019 | Rabbit | Wild | Found dead | Liver and lungs: multifocal haemorrhages | Liver: acute, severe, necrohaemorrhagic hepatitis |
| **Athl/19/01799 (2)** | 30/08/2019 | Rabbit | Wild | Found dead | Lungs: multifocal haemorrhages | Liver:acute, multifocal necrotising hepatitis; biliary coccidiosis |
| **Kilk/19/02288** | 04/09/2019 | Rabbit | Wild | Not provided | Lungs: bilateral, focally extensive haemorrhages in caudal lobes | Liver: acute, multifocal to coalescing, necrotising hepatitis; Lungs: diffuse hyperaemia and multifocal alveolar haemorrhages |
| **GVir/19/01156** | 10/09/2019 | Rabbit | Domestic | Diagnostic sample | Not applicable | Not applicable |
| **Kilk/19/02410** | 19/09/2019 | Rabbit | Domestic | Not provided; lung, liver and spleen submitted for PCR | Not applicable | Not applicable |
| **Dubl/19/00429** | 26/09/2019 | Rabbit | Wild | Young female rabbit found dead | Liver: 3-4 1mm pale raised foci. | Liver: Severe, acute, periportal necrotising |

| | | | | | | | |
|---|---|---|---|---|---|---|---|
| | | | | | | hepatitis and biliary coccidiosis. | |
| **Kilk/19/02491** | 27/09/2019 | Rabbit | Wild | Not provided | Lungs: ecchymotic haemorrhages | Liver: multifocal, primarily periportal, acute necrotising hepatitis; Lungs: mild, eosinophil-rich interstitial pneumonia | |
| **Kilk/19/02493** | 27/09/2019 | Rabbit | Wild | Not provided | Autolysed. Mucous membranes pale; blood clots in thoracic and abdominal cavities | Unsuitable (autolysis) | |
| **Dubl/19/00432** | 30/09/2019 | Rabbit | Wild | Found dead | Autolysed. Unremarkable | Unsuitable (autolysis) | |
| **Dubl/19/00438** | 01/10/2019 | Hare | Wild | Neurological signs; died shortly after being brought to veterinary surgery | Unremarkable | Liver: acute, severe, necrotising periportal hepatitis | |
| **Kilk/19/02514** | 01/10/2019 | Rabbit | Wild | Eyelids swollen with white discharge | Multifocal haemorrhage in abdominal cavity; kidneys: ecchymotic haemorrhage on surface of left kidney | Not reported | |
| **Kilk/19/02557** | 07/10/2019 | Rabbit | Wild | Not provided | Not applicable | Not applicable | |
| **Dubl/19/00450** | 08/10/2019 | Rabbit | Wild | Found dead | Autolysis; no obvious gross lesions identified | Unsuitable (autolysis) | |
| **Lime/19/02154** | 09/10/2019 | Rabbit | Wild | 2 rabbits found dead | One had caudal skull facture | Not reported | High parasite burden |
| **Cork/19/02846** | 16/10/2019 | Rabbit | Wild | Not provided | Not reported | Liver: acute, periportal and midzonal necrotising hepatitis; Lungs: interstitial | |

| | | | | | | | |
|---|---|---|---|---|---|---|---|
| | | | | | | pneumonia and haemorrhage | |
| **Athl/19/02067** | 17/10/2019 | Rabbit | Domestic | One of two rabbits to die. Both sick for one day. Anorexic, lethargic, drinking a lot | Orbital abscess | Liver: acute, necrotising, periportal hepatitis | |
| **Kilk/19/02655** | 17/10/2019 | Rabbit | Wild | Found in extremis | Multifocal serosal haemorrhages in stomach, intestines and lungs. | Liver: subacute, periportal and midzonal necrotising hepatitis; Lungs: mild to moderate interstitial pneumonia with mild, multifocal haemorrhage | |
| **Dubl/19/00465** | 18/10/2019 | Hare | Wild | Hare x 1, found dead in field at a Farm, Gorey, Co Wexford, maybe 5 days dead. | Findings is consistent with the PCR finding of Rabbit Haemorrhagic Disease. | Histopathology: Severe periportal acute hepatic necrosis. | Some biliary coccidiosis. |
| **GVir/19/01307** | 21/10/2019 | Rabbit | Domestic | Diagnostic sample | Not applicable | Not applicable | |
| **Cork/19/02898** | 22/10/2019 | Rabbit | Wild | Not provided | Not reported | Histopathology: hepatocellular degeneration and necrosis. | |
| **Kilk/19/02715 (1)** | 23/10/2019 | Rabbit | Wild | Two rabbits. RHD suspected clinically | Lungs: multifocal areas of hyperaemia | Liver: acute, moderate, multifocal necrotising hepatitis; Lungs: mild, interstitial pneumonia | |
| **Kilk/19/02715 (2)** | 23/10/2019 | Rabbit | Wild | Two rabbits. RHD suspected clinically | Unremarkable | Liver: acute, multifocal necrotising hepatitis; Lungs: mild, interstitial pneumonia; Lungs:congested and oedematous, | |

| | | | | | | | |
|---|---|---|---|---|---|---|---|
| **Kilk/19/02751** | 29/10/2019 | Hare | Wild | Found dead as part of RHD passive surveillance | Autolysis. Small volume of blood tinged froth in trachea and at nares. Congested (autolysed) liver. | Liver: sinusoidal leukocytosis | |
| **Cork/19/03020** | 01/11/2019 | Rabbit | Wild | Not provided | Not reported | Liver: acute, necrotising, hepatitis | |
| **2019–14567** | 25/11/2019 | Rabbit | Domestic | Sudden death. No signs noted | Hepatomegally; pulmonary hyperaemia and oedema with petechiation; scant, liquid intestinal contents | Liver: multifocal to coalescing necrotising hepatitis. Lung: mild peribronchial lymphocytic cuffing; congestion; oedema and haemorrhage in alveoli. Kidneys: Cortical and medullary haemorrhage. Heart: multifocal myocardial haemorrhage. Spleen: multifocal haemorrhages | Parasitology: 700 epg of strongyle eggs (borderline clinical significance). |
| **Cork/19/03579** | 16/12/2019 | Rabbit | Domestic | Second rabbit to die from group of 3 in last 36 hours; Malaise. | Slight excess serosanguinous fluid in abdominal cavity; Lungs: hyperamic and oedematous with mild, pleural petechiation | Not reported | Parasitology: coccidial oocysts in faeces (incidental finding) |
